# Acute sprint exercise transcriptome in human skeletal muscle

**DOI:** 10.1371/journal.pone.0223024

**Published:** 2019-10-24

**Authors:** Hakan Claes Rundqvist, Andreas Montelius, Ted Osterlund, Barbara Norman, Mona Esbjornsson, Eva Jansson

**Affiliations:** 1 Division of Clinical Physiology, Karolinska Institutet, Stockholm, Sweden; 2 Department of Laboratory Medicine, Karolinska Institutet, Stockholm, Sweden; 3 Department of Clinical Physiology, Karolinska University Hospital, Stockholm, Sweden; Universidad Europea de Madrid, SPAIN

## Abstract

**Aim:**

To examine global gene expression response to profound metabolic and hormonal stress induced by acute sprint exercise.

**Methods:**

Healthy men and women (n = 14) performed three all-out cycle sprints interspersed by 20 min recovery. Muscle biopsies were obtained before the first, and 2h and 20 min after last sprint. Microarray analysis was performed to analyse acute gene expression response and repeated blood samples were obtained.

**Results:**

In skeletal muscle, a set of immediate early genes, *FOS*, *NR4A3*, *MAFF*, *EGR1*, *JUNB* were markedly upregulated after sprint exercise. Gene ontology analysis from 879 differentially expressed genes revealed predicted activation of various upstream regulators and downstream biofunctions. Gene signatures predicted an enhanced turnover of skeletal muscle mass after sprint exercise and some novel induced genes such as *WNT9A*, *FZD7 and KLHL40* were presented. A substantial increase in circulating free fatty acids (FFA) was noted after sprint exercise, in parallel with upregulation of *PGC-1A* and the downstream gene *PERM1* and gene signatures predicting enhanced lipid turnover. Increase in growth hormone and insulin in blood were related to changes in gene expressions and both hormones were predicted as upstream regulators.

**Conclusion:**

This is the first study reporting global gene expression in skeletal muscle in response to acute sprint exercise and several novel findings are presented. First, in line with that muscle hypertrophy is not a typical finding after a period of sprint training, both hypertrophy and atrophy factors were regulated. Second, systemic FFA and hormonal and exposure might be involved in the sprint exercise-induced changes in gene expression.

## Introduction

One unique characteristic of acute sprint exercise is the very rapid muscle glycogen degradation and large net ATP breakdown, especially in type II skeletal muscle fibres. Following three 30-s sprints the muscle glycogen and ATP content may decrease by more than 50% in these fibres [1]. A second characteristic of sprint exercise is the pronounced endocrine stress exemplified by the increase in systemic catecholamines, growth hormone and insulin [1–3]. A third characteristic is the hyperaemic response during the post exercise period, evident as an increase in leg blood flow up to more than 30 min post sprint [4]. During the last decade, it has become apparent that sprint exercise induces health benefits similar to those of endurance training. This can be exemplified by increased aerobic capacity, insulin sensitivity, lipid oxidation capacity and vascular function, as well as decreased blood pressure and waist circumference [5].

In comparison to resistance or endurance exercise the acute signalling events that mediate skeletal muscle remodelling following sprint exercise are less known. Like resistance exercise, sprint exercise is characterised by repeated sessions of brief intermittent episodes of very high workloads. However, in contrast to resistance exercise, sprint exercise also seems to induce skeletal muscle remodelling that resembles changes usually associated with endurance training. As few as 6 sessions of sprint training over a period of 2 weeks, totalling less than 5 min of ‘all-out’ 30-s cycle exercise have been shown to increase the maximal activity of mitochondrial enzymes and to improve aerobic performance [6]. Furthermore, Gibala *et al*. [7] showed that acute bouts of sprint exercise upregulated AMPK, p38 and *PGC-1A* mRNA, which are all linked to mitochondrial biogenesis. Surprisingly, no activation of the hypertrophy signalling mTOR-pathway was demonstrated [7]. Our earlier findings of acute sprint exercise are partly consistent with this, even though we reported some increase in mTOR activation [2]. However, an increase in muscle cross-sectional area is not systematically observed in longitudinal sprint training studies [8, 9].

Moreover, there are indications that sprint exercise may counteract muscle protein accretion. For instance, Coffey *et al*. [10] reported that resistance exercise-induced Akt/mTOR signalling was inhibited when preceded by sprint exercise. It has also been shown that sprint exercise stimulates capillarisation and vascular growth in skeletal muscle in the same way as does endurance training [11], yet this observation has been questioned by others [12]. To further examine the skeletal muscle adaptations to sprint exercise, a high-throughput strategy may increase understanding of the complex exercise stimuli (such as mechanical, metabolic, neuromuscular, hormonal and substrate activation), sensed by signalling pathways, followed by activation of transcription, translation and ultimately transferred into biological functions [13, 14]. To the best of our knowledge, changes in global gene expression in skeletal muscle in response to acute bouts of sprint exercise have not been previously published. In view of the pronounced increase in exercise stimuli such as systemic growth hormone and insulin during a sprint exercise session [1–3, 13], global correlation analysis between changes in gene expression and hormonal response may add further value. Such global gene expression analyses, may increase understanding of how the health benefits of sprint exercise are regulated.

The aim of the present study was to examine the molecular response to the previously demonstrated profound metabolic, hormonal and circulatory stress responses to sprint exercise. Our approach was to measure global gene expression analysis of human skeletal muscle after repeated 30-s sprints in healthy men and women.

## Results

### Background data

The physical activity index did not differ between men and women (16±3 versus 16±1, *P* > 0.05). The men had a lower percentage of type I muscle fibres than did women (49±9 versus 63±5%, *P* < 0.005). In general, men produced higher power output values than women, both in absolute values (mean power sprint 1 and 3: 693±78 and 668±72 versus 502±80 and 486±73 W, *P* < 0.001) and related to body mass (8.7±1 and 8.0±1 versus 7.4±1 and 7.2±1, W x kg^-1^ body mass, *P* < 0.001), but not when related to fat-free body mass (9.6±1 and 9.3±1 versus 9.6±1 and 9.3±1, W x kg^-1^ fat free mass, *P* > 0.05).

### Substantial changes in blood-borne substrates, metabolites and hormones

**Fig 1:**
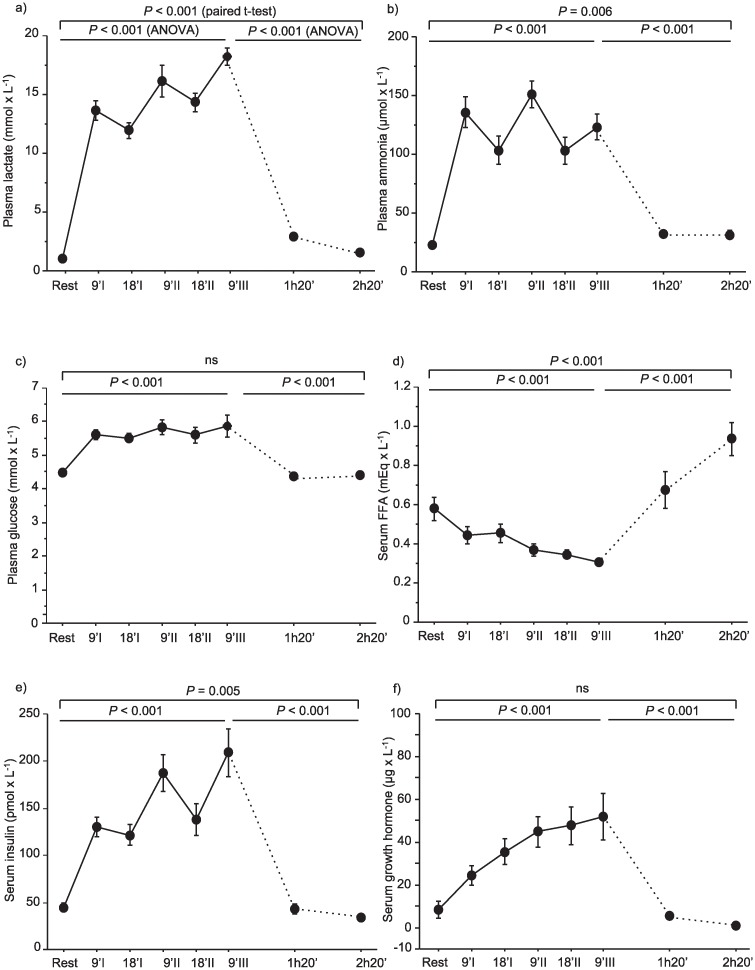
a-f. Sprint exercise-induced changes in blood-borne substrates, metabolites and hormones. Plasma concentration of (a) lactate, (b) ammonia and (c) glucose and d) serum concentration of FFA, e) insulin and f) growth hormone at rest and during the exercise period of three bouts of 30-s sprint exercise with 20-min rest in between followed by a post-exercise sampling period up to 2 h and 20 min in 7 men and 7 women. *P*-values represent statistical level of time effect of the exercise period (ANOVA) the post-exercise period (ANOVA) and of the comparison of rest and the last point of the post-exercise period (paired t-test).

Changes in blood variables are presented in Fig 1a–1f. During the sprint exercise session the concentration of serum-free fatty acids (FFA) decreased (*P* < 0.001) and was followed by an increase after the last sprint (*P* < 0.001). At the time of the post-exercise biopsy obtained 2 h 20 min after the last sprint exercise, the serum FFA level was approximately 90% higher than at rest (*P* < 0.001). Plasma lactate, plasma ammonia, serum insulin, serum glucose and serum growth hormone (GH) level increased during the sprint exercise session (all, *P* < 0.001) and was followed by a decrease after the last sprint (all, *P* < 0.001).

### Gene expression was markedly affected by sprint exercise

The PCA analysis demonstrated no systematic variation by sex/gender (S/G) in the transcriptome in the current study (S1 and S2 Figs). It was therefore justified to analyse sprint exercise-induced transcriptome changes in pooled data with regard to S/G.

Genes that were differentially expressed in muscle tissue 2 h 20 min after the last sprint were identified by gene expression array analysis. The SAM algorithm identified 879 genes, 471 being upregulated and 408 downregulated genes with fold change (FC) higher than 1.2 and false discovery rate (FDR or q-value) lower than 10%. The five ‘top-genes’ *FOS* (75-fold; expressed as 75x in the following), *NR4A3* (47x), *MAFF* (43x), *EGR1* (42x) and *JUNB* (19x) represent a set of immediate early genes (IEG). No significant changes in these five top-genes were evident in controls [15]. The top 35 upregulated genes are presented in Table 1 and all 879 genes are presented in S1 Table.

**Table 1:** Top 35 upregulated genes in skeletal muscle by sprint exercise in 7 men and 7 women.

Gene symbole	FC	FDR	Gene name	EntrezID
*FOS*	75	0.00E+00	Fos proto-oncogene, AP-1 transcription factor subunit	2353
*NR4A3*	47	0.00E+00	nuclear receptor subfamily 4 group A member 3	8013
*MAFF*	43	0.00E+00	MAF bZIP transcription factor F	23764
*EGR1*	42	0.00E+00	early growth response 1	1958
*JUNB*	19	4.04E-03	JunB proto-oncogene, AP-1 transcription factor subunit	3726
*IER2*	18	8.50E-03	immediate early response 2	9592
*OTUD1*	9.3	0.00E+00	OTU deubiquitinase 1	220213
*NR4A1*	8.9	0.00E+00	nuclear receptor subfamily 4 group A member 1	3164
*DDIT4*	8.7	4.04E-03	DNA damage inducible transcript 4	54541
*PIM1*	8.1	0.00E+00	Pim-1 proto-oncogene, serine/threonine kinase	5292
*HSPA8*	7.0	9.57E-02	heat shock protein family A (Hsp70) member 8	3312
*GEM*	6.8	5.97E-02	GTP binding protein overexpressed in skeletal muscle	2669
*CISH*	6.3	4.04E-03	cytokine inducible SH2 containing protein	1154
*GADD45B*	5.7	3.77E-02	growth arrest and DNA damage inducible beta	4616
*CCN1*	5.7	4.04E-03	cellular communication network factor 1	3491
*ANKRD1*	5.6	8.50E-03	ankyrin repeat domain 1	27063
*MYC*	5.1	0.00E+00	MYC proto-oncogene, bHLH transcription factor	4609
*PPARGC1A*	4.6	0.00E+00	PPARG coactivator 1 alpha	10891
*FOSB*	4.6	2.73E-03	FosB proto-oncogene, AP-1 transcription factor subunit	2354
*PTTG2*	4.4	0.00E+00	pituitary tumor-transforming 2	10744
*SLC16A6*	4.4	0.00E+00	solute carrier family 16 member 6	9120
*ATF3*	4.2	0.00E+00	activating transcription factor 3	467
*BHLHE40*	4.2	0.00E+00	basic helix-loop-helix family member e40	8553
*GDNF*	3.9	0.00E+00	glial cell derived neurotrophic factor	2668
*CSRNP1*	3.7	0.00E+00	cysteine and serine rich nuclear protein 1	64651
*SLC25A25*	3.7	0.00E+00	solute carrier family 25 member 25	114789
HSPA1A	3.7	0.00E+00	heat shock protein family A (Hsp70) member 1A	3303
*WNT9A*	3.7	0.00E+00	Wnt family member 9A	7483
*DUSP1*	3.6	0.00E+00	dual specificity phosphatase 1	1843
*XIRP1*	3.6	0.00E+00	xin actin binding repeat containing 1	165904
*KLHL40*	3.5	0.00E+00	kelch like family member 40	131377
*SLC20A1*	3.5	0.00E+00	solute carrier family 20 member 1	6574
*PRKAG2*	3.5	2.73E-03	protein kinase AMP-activated non-catalytic subunit gamma 2	51422
*IER5*	3.3	4.04E-03	immediate early response 5	51278
*APBB3*	3.3	0.00E+00	amyloid beta precursor protein binding family B member 3	10307

FC = fold change; FDR = false discovery rate

**Fig 2:**
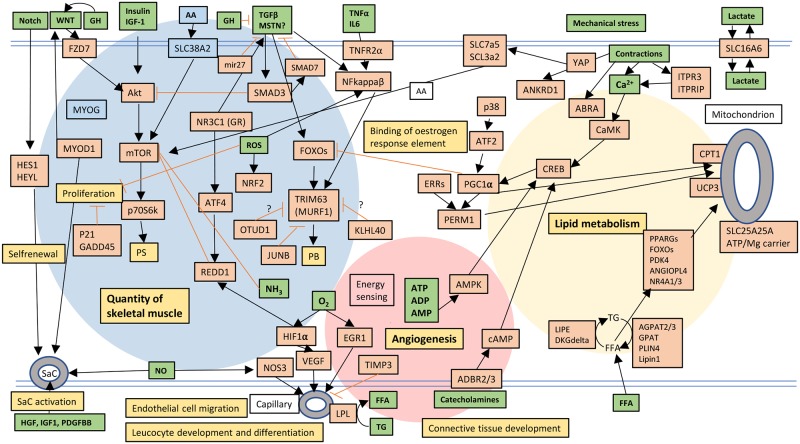
Simplified overview of molecular signalling after sprint exercise. A selection of stimuli (green–increased and blue—decreased), signalling pathways (red–upregulated and blue–downregulated and downstream biofunctions (yellow) are presented. The selection is based on skeletal muscle mRNA levels (microarray), predicted upstream regulators (IPA-analysis), predicted downstream biofunctions (IPA-analysis), protein phosphorylations (Western blot) and blood data (substrates and hormones) from the present study and earlier publications from the same experimental set up [2, 16]. AMPK activation after sprint exercise was shown by Gibala *et al* 2009 [7] and inhibition of mTOR by ammonia (NH_3_) was shown by Kumar *et al* [17] *in vitro*. Observe that it is not possible, in this figure, to differentiate between how the various pathways are activated, e.g. phosphorylations, transcriptional activation etc.) Three major areas are highlighted: regulation of muscle mass, lipid/energy metabolism and angiogenesis. For details and explanations see Results and Discussion. PS = protein synthesis, PB = protein breakdown, ROS = reactive oxygen species.

Below is an overview of differentially expressed genes using IPA analysis (canonical pathways, biological function and upstream regulators) followed by correlation analyses of gene expression versus physiological measurements (serum GH and plasma insulin) and results from validation experiments. The response to sprint exercise is also summarized in Fig 2.

### Sprint exercise-induced differential gene expression evaluated by IPA

#### Canonical pathways

In response to the acute sprint exercise, significantly increased signalling was predicted for TNFR2 (z = 2.2, *P* = 8E-03), Ceramide (z = 2.3, *P* = 1E-02), IL-6 (z = 1.8, *P* = 3E-04), NRF2 (z = 2.2, *P* = 1E-03) and p38 (z = 2, *P* = 1E-02) pathways. The pathways Adipogenesis and Molecular Mechanisms of Cancer were also predicted to be significantly regulated, (*P* = 6E-06, *P* = 1E-04), but the direction was not possible to state (z-value non-significant), (S2 Table).

#### Biofunctions

**Fig 3:**
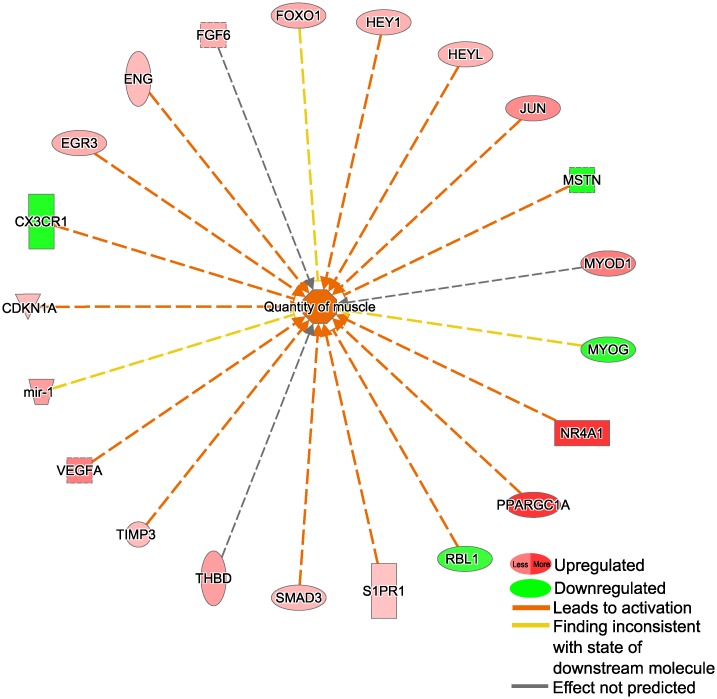
Sprint exercise—Induced changes in gene expression related downstream biofunction. Differentially expressed genes related to the predicted downstream function ‘quantity of muscle’ are depicted. The figure was created using IPA-tools.

Several increased biofunctions were identified (bias corrected z-value cut off at 1.96 and a *P*-value cut off at 1E-04, if otherwise not stated) including endothelial cell migration, differentiation and development of connective tissue, quantity of muscle (Fig 3), quantity of adipose tissue, oxidation of fatty acids (3E-04) and lipid synthesis (*P* = 1E-03). Angiogenesis was also significantly regulated (*P* = 2E-13), but the direction only tended to be positive (z = 1.7). Decreased biofunctions include inflammation in the body cavity and thrombosis. For more details see Table 2 and S3 Table.

**Table 2:** Biofunctions as identified by the IPA-analysis from 879 differentially expressed genes in skeletal muscle after sprint exercise in 7 men and 7 women. For an extended list see S3 Table.

Diseases or Functions Annotation	P-value	Predicted activation state	Activation z-value	# Molecules
cellular homeostasis	1.7E-04	Increased	4.2	132
cell survival	3.1E-05	Increased	4.2	120
differentiation of bone marrow cells	6.3E-04	Increased	3.2	19
differentiation of mononuclear leukocytes	7.7E-04	Increased	3.0	50
differentiation of lymphatic system cells	2.9E-04	Increased	2.8	21
development of mononuclear leukocytes	8.9E-04	Increased	2.7	53
development of leukocytes	2.7E-04	Increased	2.6	59
expansion of cells	1.3E-06	Increased	2.4	38
quantity of muscle	1.3E-05	Increased	2.4	19
cell cycle progression of muscle cells	7.2E-05	Increased	2.4	8
oxidation of fatty acid	3.4E-04	Increased	2.4	20
differentiation of connective tissue	1.6E-10	Increased	2.3	87
synthesis of lipid	8.1E-04	Increased	2.3	63
differentiation of stem cells	2.6E-04	Increased	2.2	28
quantity of skeletal muscle	7.3E-04	Increased	2.2	7
migration of cells	9.4E-08	Increased	2.2	171
binding of estrogen response element	9.3E-04	Increased	2.1	6
development of connective tissue	1.3E-05	Increased	2.1	45
quantity of adipose tissue	9.5E-05	Increased	2.1	31
proliferation of immune cells	1.7E-06	Increased	2.0	80
Thrombosis	1.2E-04	Decreased	-2.2	22
inflammation of body cavity	5.7E-04	Decreased	-2.3	70
Hypertension	7.8E-04	Decreased	-2.6	59

^#^ = number of molecules

#### Upstream analysis

Predicted increase in activity state was identified for 63 factors (bias corrected z-value cut off at 1.96 and a *P*-value cut off at 1E-04, if otherwise not stated). The most common type of upstream activators were transcriptions regulators (ATF2, ATF4, CREB1, CREM, EGR1, FOXO3, FOXL2, FOXO1, HIF-1α, NFKB1A, NUPRI, SMAD3, STAT3, YAP1 (*P* = 3E-03)), followed by growth factors (AGT, BMP2, EGF, HGF, IGF1, LEP, NRG1, PDGFBB, TGFβ1, TGFB3, VEGFA), cytokines (CSF3, IFNG, II3, IL1β, IL5, LIF, TNF), kinases (EGFR, IPMK, PRKCE, mTOR, Pka, Pkc(s), p38 MAPK) and hormones (βoestradiol, CAMK4, growth hormone, insulin, norepinephrine, progesterone L-triiodothyronine). In addition, some small molecules were identified as upstream regulators (calcium, fatty acids *(P* = E-02)), hydrogen peroxide, nitric oxide, quinolinic acid, (*P* = 2E-04) and as well as a few ligand-dependent nuclear receptors (ESRRG (*P* = 2E-04)), NR3C1, PGR, PPARG), peptidases (F2, F7) and transmembrane receptors (CSF2RB, IL6ST, NOTCH), see S4 Table. A predicted decrease was only identified for a few factors SCD, HDAC and COL18A1 and NONO using the cut-offs above.

### Sprint exercise affects factors related to skeletal muscle mass and activation

**Fig 4:**
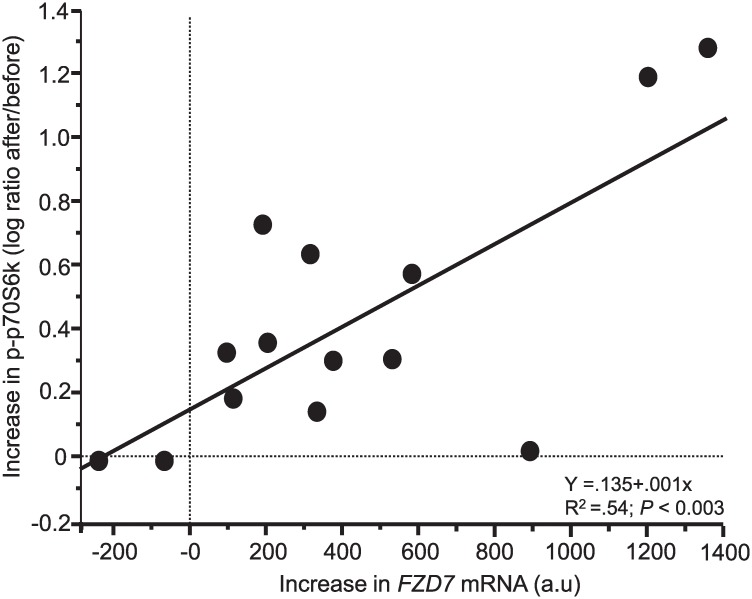
Sprint exercise–induced changes of gene expression related to changes in phosphorylation of p70S6k. Relationship between exercise-induced increase (2 h and 20 min post-exercise compared to rest) in p70S6k^thr 389^ and in mRNA of *FZD7* in 7 men and 7 women. The increase in p70S6k is expressed as logarithm of the ratio after/before.

Calcium and CaMK4 was identified as upstream regulators by IPA. Calcium-related gene expression increased such as *ITPR3* (1.3x) and *ITPRIP* (1.6x). Mechanosensitive genes such as *ANKRD1* (5.6x) and *ABRA/STARS* (1.5x) were upregulated and YAP1 was identified as an upstream regulator (Fig 2). None of these genes were changed in the controls [15]. ‘Quantity of muscle or skeletal muscle’ was a predicted biofunction by IPA (Fig 3) and mTOR was predicted to be an upstream activator after sprint exercise. The expression of *FZD7* increased (1.9x), which directly activates mTOR[18]. In the present study, a significant positive correlation (r = 0.73, *P* < 0.003) was found between the increase in phosphorylation of p70S6k, a marker for mTOR activation, and the increase in *FZD7* mRNA expression (Fig 4). Data on sprint exercise-induced phosphorylation of p70S6k have been published earlier [2], but the correlation to mRNA expression is novel and not published earlier. The gene expression of the muscle transcription factor *MYOD1* increased (2.7x), but myogenin decreased (-1.3x). A downstream target of MyoD1 [19], ‘muscle enriched gene’ KLHL40 increased (3.5x). The gene expression of the antigrowth factor *MSTN* decreased (-2.0x) and of the ‘atrogen’ *TRIM63* (*MURF1*) increased (2.4x). Some of the differentially regulated microRNAs were related to skeletal muscle mass: *mir*1 (2.0x), *mir23* (1.4x), *mir27* (1.5x), *mir29* (2.8x) and *mir95* (2.4x) [20]. None of the genes mentioned in this paragraph were differentially expressed in non-exercise controls [15].

### Correlations of sprint exercise–induced changes in gene expression and in blood hormones

**Fig 5:**
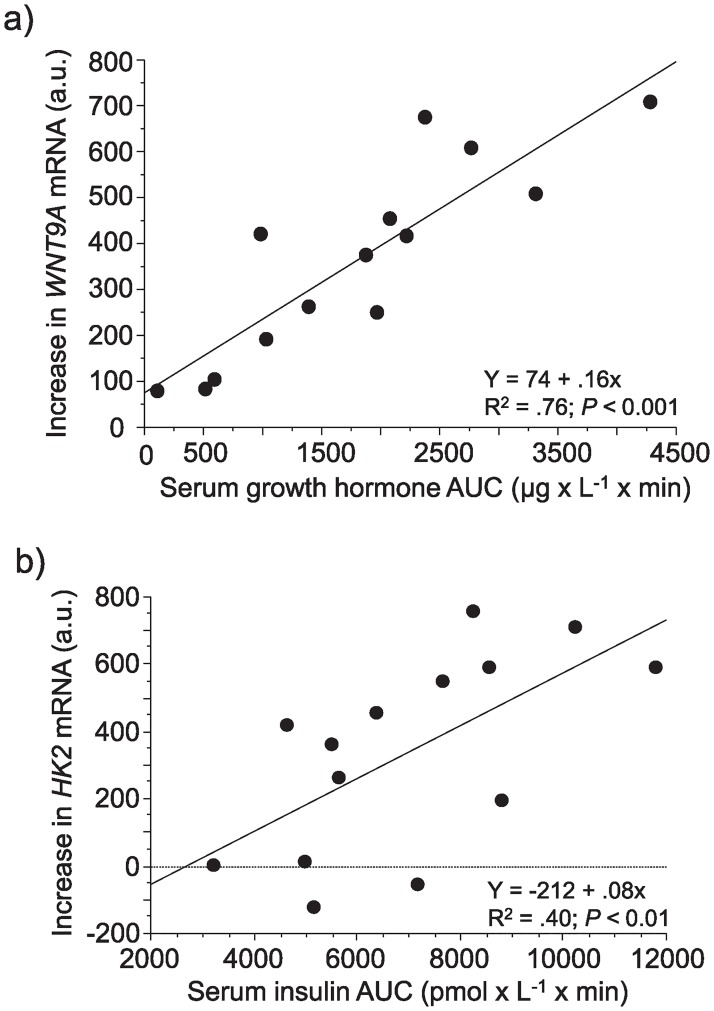
a-b. Sprint exercise—Induced changes of gene expression related to changes in hormones in blood. a) Relationship between exercise-induced increase (2 h and 20 min post-exercise compared to rest) in a) *WNT9* mRNA and AUC for serum growth hormone and in b) *HK2* mRNA and AUC for serum insulin in 7 men and 7 women. AUC = area under the curve was calculated from rest to 9 min post-exercise.

Quantitative samr was used to systematically search for relationships between sprint exercise- induced changes in gene expression and hormone exposure (estimated by area under the curve (AUC) from rest to 9 min after the last 30-s sprint). Among differentially expressed genes, some showed expression changes as exemplified by *WNT9A* (r = 0.87, FDR = 2.7%) of the Wnt signalling pathway (Fig 5a) and *FOSL2* (r = 0.84, FDR = 5.1%) of the JUN signalling pathway that both were correlated to the sprint exercise-induced increase in GH. Increase in *HK2* expression (Fig 5b) was correlated to sprint exercise-induced insulin (r = 0.64, FDR = 9.6%). Data from this study on sprint exercise-induced levels of GH and insulin have been published before (2), but data on correlations to mRNA are novel data not published earlier.

### Validation of microarray by quantitative RT-PCR

To validate the microarray data a number of genes were also analysed by RT-PCR. A positive correlation was demonstrated (r = 0.87, *P* < 0.001) when mRNA data of each gene analysed by microarray were plotted in an X-Y diagram against corresponding RT-PCR data (S3 Fig).

## Discussion

### Major findings

This is the first study, to the best of our knowledge, that comprehensively presents changes in global gene expression in skeletal muscle in response to acute bouts of sprint exercise, a type of exercise known to induce a profound local and systemic stress [1–3]. One novel finding was that acute bouts of sprint exercise induced an approximately 20-75-fold expression of a subset of immediate early genes (IEG). Other novel findings were that gene signatures predicted activation of various exercise stimuli such as Ca2^+^, NO, ROS, and also of exercise-induced systemic factors such as growth hormone, insulin and fatty acids (Fig 2). Levels of these systemic factors were also markedly increased in blood during the exercise session or post exercise. Downstream gene expressions predicted an enhanced lipid turnover and genes such as *PGC-1A and PERM1* were upregulated together with a large number of genes directly related to lipid metabolism. Gene signatures also predicted activation of muscle quantity. The mRNA expression of *FZD7*, a positive regulator of Akt/mTOR and muscle mass, was enhanced after sprint exercise and correlated to the increase in phosphorylation of p70S6k in the mTOR-pathway. The exercise-induced increase in *WNT9A* mRNA, a potential upstream activator of *FZD7* and known to be regulated by GH [21], was related to the increase in serum GH. However, sprint exercise also affected several genes in the direction of atrophy. In addition, expression of two novel genes with possible anti-atrogen function, *KLHL40* and *OTUD1*, was increased at the mRNA level after sprint exercise [22, 23]. Finally, gene signatures indicated an activation of extracellular matrix remodelling related to angiogenesis.

### Sprint exercise markedly upregulates immediately early genes

One of the striking findings was that acute bouts of sprint exercise induced an approximately 20-75-fold expression of a subset of IEG. One of the top-5 upregulated genes in the current study, *EGR1* (42x), was also identified as an upstream regulator and is described in the literature as a ‘master regulatory’ transcription factor [24]. EGR1 is involved in processes such as mitochondrial biogenesis [25] and angiogenesis [24] and like HIF-1α, ERG1 is described as a hypoxia-inducible proangiogenic factor [24, 26], although less is known about its significance in human skeletal muscle. Recently, however, EGR1was suggested to be a key factor involved in a coordinated cycle through which exercise and insulin sensitivity regulate gene expression in skeletal muscle as based on an experimental human microarray study [27]. In concordance with our findings, Edgett *et al*. [28] found a 40-fold increase in *EGR1* mRNA after high intensity exercise and concluded that increase in *EGR1* mRNA was more dependent on exercise intensity than e.g. *PGC-1A* mRNA and its regulators.

### Sprint exercise stimuli and related predicted signalling

#### Various stimuli

As previously mentioned, a total of 167 upstream regulators were identified (S4 Table). Examples of stimuli as identified by upstream regulators in the IPA analysis are Ca2^+^, CaMK4 (neuromuscular); nitric oxide, hydrogen peroxide, HIF-1α and p38 (metabolic); oestrogen, GH, insulin, noradrenaline and T3 (hormonal).

#### Ca2^+^ signalling

Of special interest was the finding that sprint exercise induced changes in expression of the IP3 receptor *ITPR3* mRNA and also of *ITPRIP* mRNA, which has been shown to modulate IP3-receptor sensitivity for calcium [29]. IP3-induced calcium transients activate ERK and CREB in rat myotubes and increase IEG mRNAs such as *c-FOS*, *c-JUN* and *EGR1* [30, 31]. After sprint exercise in the current study, both ERK and CREB were predicted to be activated together with a profound increase in IEG mRNAs.

#### Mechanical stimulus

YAP1 is mechanosensitive transcriptional cofactor and has earlier been reported to increase the expression of the amino acid (AA) transporters *SCLA7a5* and *SCL3a2* [32], which in turn may activate mTOR. In the present study YAP was identified as an upstream regulator after sprint exercise and the downstream AA transporters *SLC7a5* and *SLC3a2* were upregulated at the mRNA level. In addition, *ANKRD*1, another downstream target of YAP, located in the sarcomere region [33], was upregulated after sprint exercise. Finally, *ABRA/STARS*, with a possible mechanosensitive function, was also upregulated at the mRNA level following sprint exercise [34].

#### *Metabolic stimulus*—*Upstream and downstream of* PGC-1α

*PPARGC1A* (*PGC-1A)* expression was upregulated 5-fold by sprint exercise. Possible upstream regulators identified from the literature such as Ca2^+^, p-38, ATF2, CREB, ADRB3 and ERRG [35–37] were also identified in the present study. Downstream targets thought to be regulated by PGC-1α [38–40] were also identified and exemplified by *PPARD*, PPARG (upstream regulator), *PDK4* and *VEGF* as well as *ABRA*, *PERM1* (PGC-1α and ERR-induced regulator in muscle 1) and lipin1, [34, 41, 42]. An exercise-induced increase in *PERM1* mRNA, encoding for a muscle-enriched protein, has, to the best of our knowledge, only been reported once before and was found to stimulate mitochondrial biogenesis [41, 42]. However, no mitochondrial transcription factors were found to be upregulated after sprint exercise, except for mitochondrial transporters, the ATP/Mg^2+^/phosphate carrier *SLC25A25A*, the carnitine carrier *CPT1* and the proton carrier *UCP3*. Increased *PCG-1A* mRNA level seems to be a general response to various exercise modalities such as endurance [15, 43] and resistance exercise [15, 44, 45].

#### Oestrogen receptors and predicted activation

In the current study the oestrogen response element (ERE) was predicted to be activated, and oestrogen related receptor gamma (ESRRG) and oestrogen were suggested to be upstream regulators. This agrees with an earlier study in myotubes suggesting that both muscle contractions and oestrogen activate ERE [46]. The upregulation of *PERM1*, mentioned above, is of special interest because it is a downstream target of both ERR and PGC-1α.

#### Substrate activation

Serum FFA concentration increased markedly after sprint exercise in the present study and cannot be excluded to be a stimulus for the increase of some of the metabolic genes. This is collectively supported by data from the literature. Firstly, *ANGPL4*, *KLF10* and *PDK4* were also upregulated in skeletal muscle of controls after 2.5 h of fasting [15], a condition supposed to increase circulating FFA. Secondly, a one-leg endurance exercise study showed that *ANGPL4*, *KLF10 and PDK4* mRNA expression, increased in both the exercised and the non-exercised leg, suggesting non-contractile systemic factors being involved [47]. Finally, some sprint exercise-induced genes (e.g. *ANGPL4* and *PPARD*) are known to be transcriptionally regulated by FFA [47–49].

#### Hormonal stimuli

Several hormones or related substances were predicted to be upstream regulators (glucocorticoids, GH, insulin, noradrenaline, oestradiol and T3) and some of these hormones (GH, insulin and noradrenaline) are also known to markedly increase in blood in response to sprint exercise [2, 16]. Correlations between increase of GH and insulin in blood on the one hand and increase in known hormone gene targets such as *WNT9A* and *HK2* mRNA on the other hand, support such predictions.

### Sprint exercise affects counteracting factors related to skeletal muscle mass

#### Myostatin, related genes and upstream regulators

Myostatin (MSTN), a powerful negative regulator of muscle mass [50] was the most downregulated gene in the current study (-50%). A downregulated *MSTN* expression may thus favour an increase in muscle mass. Upregulation of MYOD1 and *HES1* as well as the identification of GH as an upstream regulator together with the exercise-induced increase in GH in blood in the current study are all in concordance with an inhibition of MSTN signalling and gene expression [51–54]. In contrast, some downstream genes indicated an activation of MSTN signalling: *CDKN1A/p-21* (upregulated), myogenin (downregulated), *SMAD*3 (upregulated) and *TRIM63* (upregulated) together with the predicted upstream regulators TGFβ, SMAD3 and FOXO3. However, the net result of up- and down-regulators of MSTN signalling was not possible to state in the present study, in spite of the decreased mRNA level (*P* = E-03, z = 1.0). Interestingly, *SMAD7* mRNA and *miR27* were upregulated and both of these inhibit *MSTN* gene expression by negative auto-regulation [55, 56]. The downregulation of *MSTN* mRNA seems to be a general response to various exercise modalities such as endurance and resistance exercise [15, 43, 44] and also a response to exogenous growth hormone exposure [21]. As earlier suggested [57] the downregulation of *MSTN* mRNA after exercise could partly be a result of a negative feedback regulation.

#### Upstream regulators of mTOR-signalling

mTOR was predicted by the IPA-tool to be activated after sprint exercise and has previously been reported to be weakly activated as demonstrated by Western blotting [2]. In the present study FZD7, an upstream regulator of Akt/mTOR signalling and muscle mass [18], was upregulated at the mRNA level and positively correlated to the increase in phosphorylated p70S6k, a downstream marker of mTOR signalling. *WNT9A* mRNA was also upregulated in the present study and associated with the exercise-induced increase in serum GH. Interestingly, Wnt signalling has been shown *in vitro* to be an upstream activator of FZD7 [18, 58]. In addition, *MYOD*1 mRNA was increased by sprint exercise and is known to upregulate *WNT9A* [59]. Finally, it was recently demonstrated [60] that hypoxia activates MYOD1, which in turn activates non-canonical Wnt signalling (e.g. through WNT9A), resulting in myotube hypertrophy. The sprint exercise response of MYOD1, WNT9A and FZD7 the mRNA level is shared by resistance exercise [15, 44] and GH exposure [21].

#### Factors related to ‘atrophy signalling’

Sprint exercise also affected several genes related to inhibition of protein synthesis or activation of protein breakdown that may counteract the hypertrophy signalling, even though some of these changes might reflect a normal remodelling process [61]. Such activation of counteracting factors is supported by that muscle hypertrophy is not a typical finding after a period of sprint training [8, 9]. Examples of affected genes related to atrophy signalling are the leucine transporter *SLC38A2* mRNA (*SNAT2*) that decreased and *SMAD3*, *REDD1*, *GADD45a* and the atrogen *TRIM63* mRNA (*MURF1*) that all increased. In addition, several upstream regulators related to muscle atrophy were predicted to be activated by sprint exercise: e.g. the glucocorticoid receptor atf4 [62], AMPK, PRKAG2, TGFβ, SMAD3 and FOXO1-3 [63, 64]. By the present global gene expression analysis, however, it was not possible to predict a direction of net change in muscle mass by sprint exercise. Several genes were thus affected in the direction of atrophy, reducing the result of a potential hypertrophy process, yet this may also reflect a normal remodelling process. The interpretation of the predicted activation of ‘muscle quantity’ by the IPA-tool is not obvious.

#### Factors that may counteract atrogens

After both sprint and resistance exercise [15] the deubiquitinase, *OTUD1* mRNA was upregulated. OTUD1 removes ubiquitin molecules and has a probable signalling regulatory role [22]. However, it is unknown if OTUD1 is involved in deubiquitination and stabilization of contractile proteins. *KLHL40* was also upregulated after both sprint and resistance exercise and has been shown to stabilize nebulin by blocking ubiquitination [23]. Finally, JunB that was upregulated after sprint exercise promotes hypertrophy by block the binding of FOXO to atrogenes [65].

#### Cell cycle

Several cell cycle regulators were markedly upregulated (*FOS*, *JUN*, *MYC*) but also some cell cycle inhibitors (*CDKN1A/p-21*, *GADD45*). In the IPA analysis this was indicated by a strong regulation of the pathway ‘Molecular Mechanisms of Cancer’, but no significant direction was possible to identify. As discussed by Chen *et al*. [66], a simultaneous upregulation of both cell cycle activators and inhibitors might be necessary in order to keep the muscle cell nuclei in the post-mitotic phase. Signs of sprint exercise regulation of satellite cell and myoblast cell cycle were also apparent. *MSTN* expression was downregulated and the Notch downstream targets *HES1* and *HEYL* were upregulated, as was *MYOD1*, but myogenin was downregulated. The net effect of these changes is not possible to clearly state, even though this may indicate an enhanced self-renewal and proliferation, but not unequivocally differentiation [67].

### Sprint increases post-exercise circulating FFA in parallel to gene signatures that predict enhanced lipid turnover

A novel finding was that serum FFA concentration increased markedly after sprint exercise in parallel to that oxidation of fatty acids and synthesis of lipids were predicted to increase by differentially expressed genes. These mRNAs representing fatty acid metabolism, transport, oxidation and also intramuscular TG (IMTG) metabolism and could be exemplified by *CPT1* and *SLC22A5* (carnitine transport), *GPAT*, *AGPAT2*, *AGPAT3*, *PLIN4*, lipin1 (IMTG synthesis), *DGKdelta* (IMTG breakdown) as well as *PRKAG2*, *PPARD*, *FOXO1*, *ANGPL4*, *PDK4*, *UCP3*, *NR4A1* and *NR4A3* (fatty acid oxidation). The fatty acids that are metabolised in skeletal muscle are mainly derived from the circulating FFA pool, which in turn is derived from white adipose tissue. The muscle tissue is also supplied by fatty acids from plasma lipids. The increased LPL mRNA in the present study may indicate an increased capacity to utilize plasma lipids. IMTG stores are another source for fatty acids in muscle tissue and the increased *LIPE* mRNA (1.5x; *P* = 0.001), also called hormone sensitive lipase, may indicate an increased capacity to utilize IMTG. Collectively, all these sprint exercise-induced changes in fat metabolism-related mRNAs most likely indicate an increased usage of fat as energy substrate and an increased turnover of IMTG after the sprint exercise in order to spare blood glucose. Others have demonstrated that the respiratory exchange ratio is decreased in the early post-exercise period after sprint exercise, supporting the shift towards fat metabolism [68]. It is important to note that the acute changes in fat metabolism might be induced shortly *after* the sprint exercise session because the serum FFA level decreased markedly during this period, and first increased later during the post exercise period. Further support for an activated lipid metabolism after sprint exercise was demonstrated by Burgomaster *et al*. [69], who found that 6 weeks of sprint interval training increased skeletal muscle enzymes representing fatty acid oxidation. After endurance exercise, a shift towards fat metabolism in a microarray experiment in human subjects was reported [70]. A shift towards fat metabolism may therefore be a general feature after exercise. However, it is important to acknowledge that the acute adaptations after sprint exercise probably would differ if the subjects were supplied with nutrients [71, 72].

### Sprint affects counteracting factors related to angiogenesis and remodelling

In the present study angiogenesis was strongly predicted to be regulated by acute sprint exercise even though the direction just tended to be positive (z = 1.7). However, typical for the angiogenic process is that it is regulated by both angiogenic and antiangiogenic factors and that there is a need for a balance between these factors to successfully develop the capillary system [73–75]. Other predicted functions supporting a stimulated angiogenesis were functions such as cell expansion, cell migration, endothelial cell migration, differentiation and development of various blood-borne cells such as mononuclear leucocytes, lymphocytes and bone marrow cells, differentiation and development of connective tissue. Some of the differentially expressed genes were *VEGF* and *NOS3* with angiogenic effects, and *TIMP3* with an antiangiogenic effect and e.g. HIF-1α, VEGF, PDGF BB, SP1 and EPAS1 were identified as upstream regulators. *EGR1* was also differentially expressed and predicted as an upstream regulator and has in addition to HIF-1α been suggested to respond to hypoxia and to induce angiogenesis [24, 26]. There are only a few studies exploring the chronic effects of sprint exercise or other forms of high intensity training on capillarisation in skeletal muscle, and the outcome is equivocal [11, 12].

### Acute *vs* chronic effects of sprint exercise on gene expression

Recently, data of global gene expression from an alternative form of low volume-high intensity intermittent exercise training over a period of 6 weeks were presented (6–7 sets of 20-s cycling periods at 170% of maximal oxygen uptake interspersed by 10-s rests, 4d/week) [76]. The authors identified 159 regulated genes and the enriched categories were glucose metabolism, extracellular matrix, angiogenesis and mitochondrial membranes, thus exhibiting great similarities to the findings in the present study. Interestingly, they highlighted four factors not linked to exercise before that were upregulated both at the gene and protein levels. Two of these, *SGK1* (may regulate insulin sensitivity) and *PPP1R3C* (may regulated glycogen synthesis) were also upregulated after acute exercise in the present study.

### Strengths and limitations

In light of our earlier findings of S/G related differences in sprint exercise-induced changes in blood and skeletal muscle variables [2, 3, 16], it seems relevant to search for related S/G differences in sprint exercise-induced changes in skeletal muscle gene signatures. The study included an equal number of men and women. However, due to the low statistical power for such analysis after correction for multiple comparisons in order to apply stringent criteria for FDR [77], no split by S/G was performed in the current study. The main focus was to examine the acute effect of sprint exercise *per se* on the sprint exercise gene signature as this has not been presented earlier, and the inclusion of both men and women gives the overall results better generalizability. A strength of the present study is that pre- and post-exercise biopsies were taken in contralateral legs and only one post-exercise biopsy was taken (approximately 3 hours after the first sprint) to avoid damaging effects of repeated muscle biopsies [78–81]. This is at the same time a limitation due to the fact that a single post-exercise biopsy provides only a snapshot of the exercise-induced changes in gene expression. However, the chosen time point of approximately 3 hours is commonly used in studies of acute changes in gene expression, since time course studies reveal that a large number of regulated genes peak between 2–8 hours after exercise or other exposure. The chosen point of time for sampling biopsies is therefore not that critical in this ‘early’ post exercise phase [15, 78]. Another limitation in the present study is the lack of a non-exercised control group. Such a control may adjust for changes in gene expression due to other factors than exercise *per se*, such as fasting and time of the day. To compensate for this lack of a control group the data in the current study were compared to a published data set from a control group of subjects of similar age in which two biopsies were performed with an interval of 2.5 h in a non-exercised and fasting condition [15]. A third limitation is that no methods were applied for studying the specific localisation of the differentially expressed genes within the muscle tissue. Of interest would be to study possible localisations to other cell types than the muscle fibres such as endothelial cells, fibroblasts or infiltrating blood- borne cells.

### Conclusions

Acute bouts of 30-s sprint exercise in men and women resulted in profound local and systemic stress, identified by analyses of blood and of the global transcriptome in skeletal muscle. Several novel findings are presented related to sprint exercise regulated genes. Firstly, a set of immediate early genes (IEG), increased 20-75-fold. Secondly, in agreement with that muscle hypertrophy is not a typical finding after a period of sprint training, both hypertrophy and atrophy factors (gene expression and predicted regulators) were regulated. Thirdly, hormonal exposure might be involved in the sprint exercise-induced changes in the transcriptome, as based on the pronounced increase in blood levels of GH and insulin, also related to changes in gene expressions. Moreover, both GH and insulin were predicted as upstream regulators as based on the differentially expressed genes. Fourthly, substrate activation by FFA could be a potential regulator for lipid turnover genes after sprint exercise as based on the markedly post exercise increase of FFA in blood, the predicted activation of fatty acids as an upstream regulator and the downstream prediction of enhanced lipid turnover.

## Materials and methods

### Subject characteristics

Fourteen subjects volunteered for the present study (7 men and 7 women) and age, height, body mass, relative body fat, fat free mass and BMI were 26±4 versus 25±2 year (*P* > 0.05), 182±4 versus 172±9 cm (*P* < 0.05), 83±8 versus 68±12 kg body mass (*P* < 0.05), 13±3 versus 22±5%, 72±7 versus 52±6 kg fat free body mass (*P* < 001) and 25±2 versus 23±2 kg x m^-2^ (*P* > 0.05) respectively. The inclusion criteria were: good health, leisure-time sports participation but not at an elite level and age 20–30 years. The exclusion criteria were chronic disease, acute infection, pregnancy, severe asthma or use of products containing nicotine. Physical activity level during leisure time was estimated by a questionnaire and expressed as an activity index (range 5.5–20.5) [82]. Fat-free body mass was estimated from skin fold measurements. Women in various phases of the menstrual cycle on or off contraceptives were selected study instead of selecting women in a certain phase of the menstrual cycle to get a more representative sample. All subjects were fully informed about the procedures and potential risks of the experiment before giving their consent to participate. For a full description of the methods, see an earlier publication from the current experimental study, including data on background characteristics, sprint performance, blood (glucose, lactate, ammonia, GH and insulin) and muscle proteins (e.g. mTOR signalling) [2]. Some of the data (blood and p70S6k) have been published earlier [2]. However, the blood data in previous paper [2] were presented separately for each sex/gender. No such split was made in the present paper, and the data was based on fewer subjects than in the previous publication [2]. Moreover, the blood and p70S6k data were used mainly to perform correlations with gene expressions, i.e. to generate novel findings. The data on free fatty acids have not been published earlier nor any of the gene expression data. The study was approved by the Regional Ethical Review Board in Stockholm, Sweden.

### Experimental protocol

**Fig 6:**
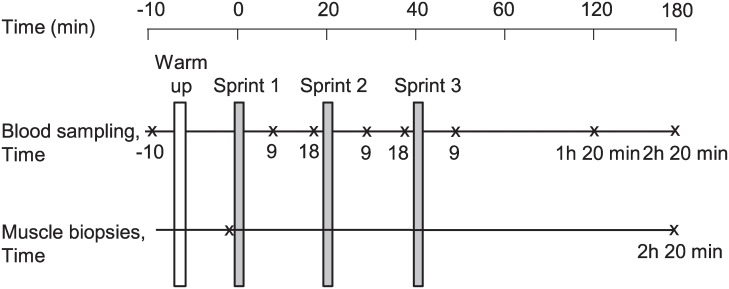
Schematic presentation of the experimental protocol. The time scale during the three sprints including 20 min rest differs from the time scale after the last sprint.

The subjects reported to the laboratory in the morning after an overnight fast. The subjects were asked to refrain from heavy exercise 48 h before the participation in the current experiment. After warm-up (1 min ergometer cycling, 60W), three all-out cycle sprints of 30-s duration with a braking load of 0.075 kp per kg body weight[83], were performed on a mechanically braked cycle ergometer (Cardionics, Bandhagen, Sweden) with 20 min of rest between sprints, (Fig 6). The reason for the 20 min of rest was to get a close-to-full recovery of the power output and the capacity for anaerobic ATP regeneration. Peak power (i.e. the highest 5-s power) and mean power (the average power during the 30-s) were calculated. Blood samples were collected from an indwelling catheter placed in an antecubital vein in the subject's forearm. With the subject in the supine position, blood was sampled before the first sprint, at various points of time during the sprint exercise session and up to 2 h and 20 min after the last sprint (Fig 6). Two biopsies were performed in the quadriceps femoris muscle (vastus lateralis) by a percutaneous needle technique under local anaesthesia without adrenaline [84]. The first biopsy sample was obtained randomly in either right or left leg before the first sprint. The second sample was obtained 2 h and 20 min after the third sprint from the contralateral leg. The samples were frozen in liquid nitrogen immediately after excision and stored at—80°C for later analyses.

### Blood sample preparation and analyses

Blood sample preparation and analyses except for non-esterified fatty acids have been described earlier in detail [2]. Briefly, plasma lactate was analysed by a Radiometer ABL 800 Flex blood gas analyser (Berman & Beving Lab, Triolab, Gothenburg, Sweden), serum insulin by an electrochemiluminescence immunoassay (Modular E170, Roche, Pharma, Stockholm, Sweden), serum growth hormone (GH) by the Immulite 2000 Growth Hormone (hGH) chemiluminescent enzyme immunoassay (Diagnostic Products Corporation, Germany), plasma glucose by an enzymatic method on a Beckman-Coulter (LX-20), plasma ammonia by an enzymatic method [85] with some modifications and serum non-esterified fatty acid (FFA) by an enzymatic colorimetric assay (Wako Chemicals GmbH).

### Biopsy sample preparation and analyses

Muscle samples obtained before exercise (50–100 mg) were immediately blotted and divided into three parts. Two parts used for either gene or protein expression analyses, were frozen in liquid nitrogen. The remaining third part used for fibre type histochemistry was frozen in isopentane cooled to its freezing point by liquid nitrogen [86]. Muscle samples obtained after exercise (30–100 mg) were immediately blotted and frozen in liquid nitrogen. All samples were stored at –80° C until processed. At the time for analysis, the frozen samples were divided into two parts, one for gene and the other for protein expression. For gene expression analyses, RNA was isolated from frozen biopsies in all 14 subjects before and after exercise. The tissue samples (10–20 mg) were treated with a homogenizing dispenser (Polytron, Kinematica) and a standard TRIzol^®^ protocol (InvitrogenTM Life Technologies, Carlsbad, CA, USA). An additional purification was performed using RNeasy Mini Kit (Qiagen, cat. No.74104). RNA was quantified using Nanodrop (Spectrometer ND-1000: NanoDrop^®^; Wender Aveen) and the RNA quality was assessed by an Agilent 2100 Bioanalyzer (Agilent Technologies, Santa Clara, CA, USA). RNA integrity values (RIN) ranged from 7.5–8.5 indicating an even and acceptable RNA quality among samples. Methods and data of protein analyses are mainly presented elsewhere [2].

### Microarray analysis and related statistics

Microarray analysis was performed using the Human Gene 1.0 ST Array (Affymetrix, Santa Clara, CA), which includes 28.869 probesets. The Affymetrix Gene Chip Whole Transcript (WT) Sense Target Labeling Assay Manual was used for complementary DNA (cDNA) generation, hybridization and array processing. In brief, from 100 ng total RNA, double-stranded cDNA was synthesized and further amplified with T7 RNA polymerase to generate complimentary anti sense RNA (cRNA). In the second-cycle cDNA synthesis, single-stranded sense-strand DNA was generated. cDNA was fragmented, biotin-labeled and hybridized to the Human Gene 1.0 ST Arrays were hybridized for 16 hours at 45°C in a Gene Chip Oven 640 (Affymetrix) followed by washing and staining using the Gene Chip Fluidics Station 450. Scanning was carried out using the Affymetrix Gene Chip Scanner 3000 7G. Raw intensity data (CEL files) were processed by using Affymetrix Expression Console Software (v.1.0). Background correction (PM-6CBG), global median normalization and summarization of probe intensity (Plier) were performed. RNA quality was analysed by RNA degradation plot. Differentially expressed genes in response to exercise were identified using the samr package in R[87]. Cut offs were applied at fold change (FC) 1.2 and false discovery rate (FDR or q-value) 10%, resulting in 471 upregulated and 408 downregulated genes. Principal component analysis (PCA) was performed to look for sex/gender (S/G) related systematic variation in the transcriptome data before and after exercise. QIAGEN’s Ingenuity^®^ Pathway Analysis (IPA^®^, QIAGEN Redwood, CA) was used to identify pathways that were significantly enriched in the list of differentially expressed genes. The array Human gene 1.0 ST was chosen as reference. Differentially expressed genes were also correlated to changes in physiological variables such as levels of hormones, lactate and FFA in blood and muscle fibre type percentages by using the samr package with the response type ‘quantitative’ and corrections were made for multiple comparisons. All raw data (CEL files) have been deposited in NCBI's Gene Expression Omnibus [88] and are accessible through GEO Series accession number GSE126296.

### Validation by quantitative real-time PCR

To validate microarray data, one microgram of total RNA was reverse transcribed using transcription kits from Applied Biosystems (High-capacity cDNA reverse Superscript) and random hexamer primers (Roche Diagnostics GmbH, Mannheim, Germany) in a total volume of 20 μL Quantitative real-time PCR (RT-PCR) was used to measure mRNA expression on an ABI-PRISMA 7700 Sequence Detector (Applied Biosystems Inc., Foster City, CA, USA). Primers and probes were supplied as a TaqMan^®^ Reagents kit (Applied Biosystems): *ACVR2B* (Hs00609603_m1), *ACTN*3 (Hs00153812_m1), *DDIT4* (Hs01111686_g1), *FBXO32* (Hs00369714_m1), *FZD*7 (Hs00275833_s1), IGF1 (Hs01547656_m1), *MSTN* (Hs00193363_m1), *MYC* (Hs00905030_m1), MYOG (Hs01072232_m1), *MYOD1* (Hs02330075_g1), *OTUD1*, (Hs02596821_s1), *PGC-1A* (Hs01016719_m1), *RHEB* (Hs00950800_m1), *SLC7A5* (Hs00185826_m1), *SLC3A2* (Hs00374243_m1), *SLC38A2* (Hs01089954_m1), *SMAD7* (Hs00998193_m1), TRIM63 (Hs00822397_m1) and *PIK3C3* (Hs00176908_m1). *RPS18* (Hs01375212_g1) was used as an endogenous control to correct for potential variation in RNA loading. All reactions were performed in 96-well MicroAmp Optical plates (Applied Biosystems). Amplification reagents (25 μL) contained the sample of 5 μL cDNA diluted 1:100 and TaqMan Universal PCR Mastermix. For each individual, all samples were simultaneously analysed in duplicate in one assay run. Measurements of the relative expression were performed for each individual; a CT value was obtained by subtracting *RPS18* CT values from respective target CT values. The expression of each target was then evaluated by 2^−ΔΔCT^ and fold changes were calculated for each gene [89].

### Analysis of published non-exercise microarray data

The list of differentially genes in the present sprint study was compared to a list of genes from a ‘control’ experiment in order to find overlapping genes. Biopsies were performed with an interval of 2.5 h in a non-exercised and fasting condition [15]. We obtained normalized and processed data files from Vissing *et al* [15] and cut-off values for fold change and FDR were chosen to be 1.2 and 10%, i.e. the same as in the present study. Thirty-nine upregulated and 52 downregulated genes were identified. The selected study was chosen to match the study populations as far as possible with regard to subject characteristics (6 healthy young men), the time interval between the biopsies (2.5 h) and array platform (Affymetrix Human Gene 1.0 ST). Statistical significance of overlap was analysed by using the hypergeometric test. Seven genes were upregulated and 14 genes were downregulated in common between controls and sprint exercise (S5 Table).

### Statistics

Values in the text are means ± SD unless otherwise stated. The *P* values were accepted as statistically significant at the level *P* < 0.05. Student's t-tests for unpaired observations were used to test sex differences in background variables (Table 1). For changes by time, a one-way ANOVA (repeated measures design; time) was employed for the blood variables and correlations were calculated by Pearson's correlation coefficient. Statistics for microarray are given in earlier paragraphs.

## Supporting information

S1 Fig PCA preexercise men and women. (PDF)

S2 Fig PCA postexercise men and women. (PDF)

S3 Fig Microarray vs PCR. (PDF)

S1 Table Differentially expressed genes. (XLSX)

S2 Table Pathways. (XLSX)

S3 Table Biofunctions. (XLSX)

S4 Table Upstream analysis. (XLSX)

S5 Table Controls. (XLSX)
