# Supplementary material for: Acute sprint exercise transcriptome in human skeletal muscle
Source: PLoS One. 2019 Oct 24;14(10):e0223024. doi: 10.1371/journal.pone.0223024 (PMC6812755; doi:10.1371/journal.pone.0223024)
Supplement: S2 Fig — (PDF) [file pone.0223024.s002.pdf]

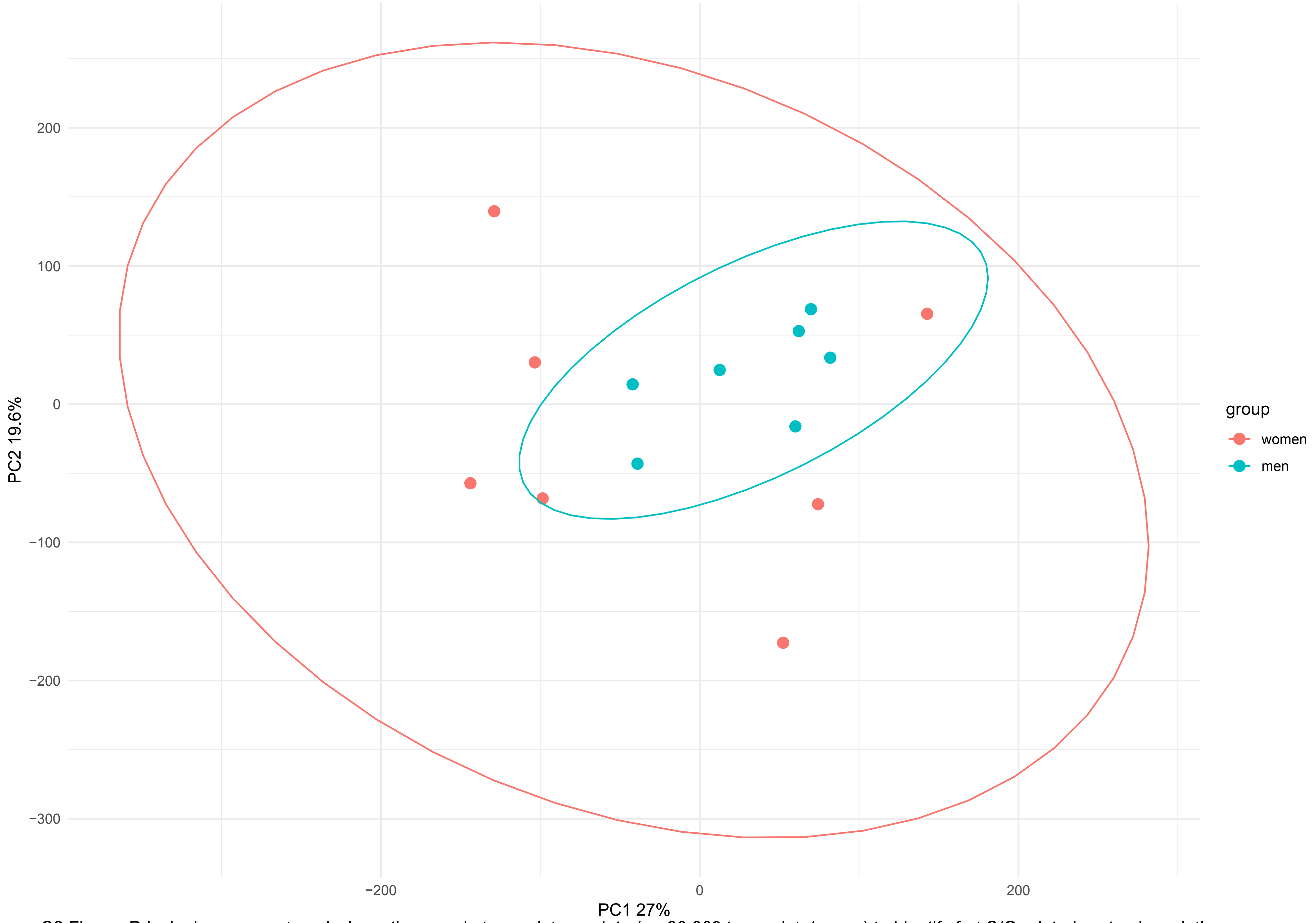

S2 Figure. Principal component analysis on the muscle transcriptome data (n= 28.869 transcripts/genes) to identify fort S/G related systemic variation after sprint exercise (postexercise) in 7 men (blue) and 7 women (red). 95% confidence intervals for each group are indicated by ellipses.
