# Supplementary material for: Acute sprint exercise transcriptome in human skeletal muscle
Source: PLoS One. 2019 Oct 24;14(10):e0223024. doi: 10.1371/journal.pone.0223024 (PMC6812755; doi:10.1371/journal.pone.0223024)
Supplement: S3 Fig — (PDF) [file pone.0223024.s003.pdf]

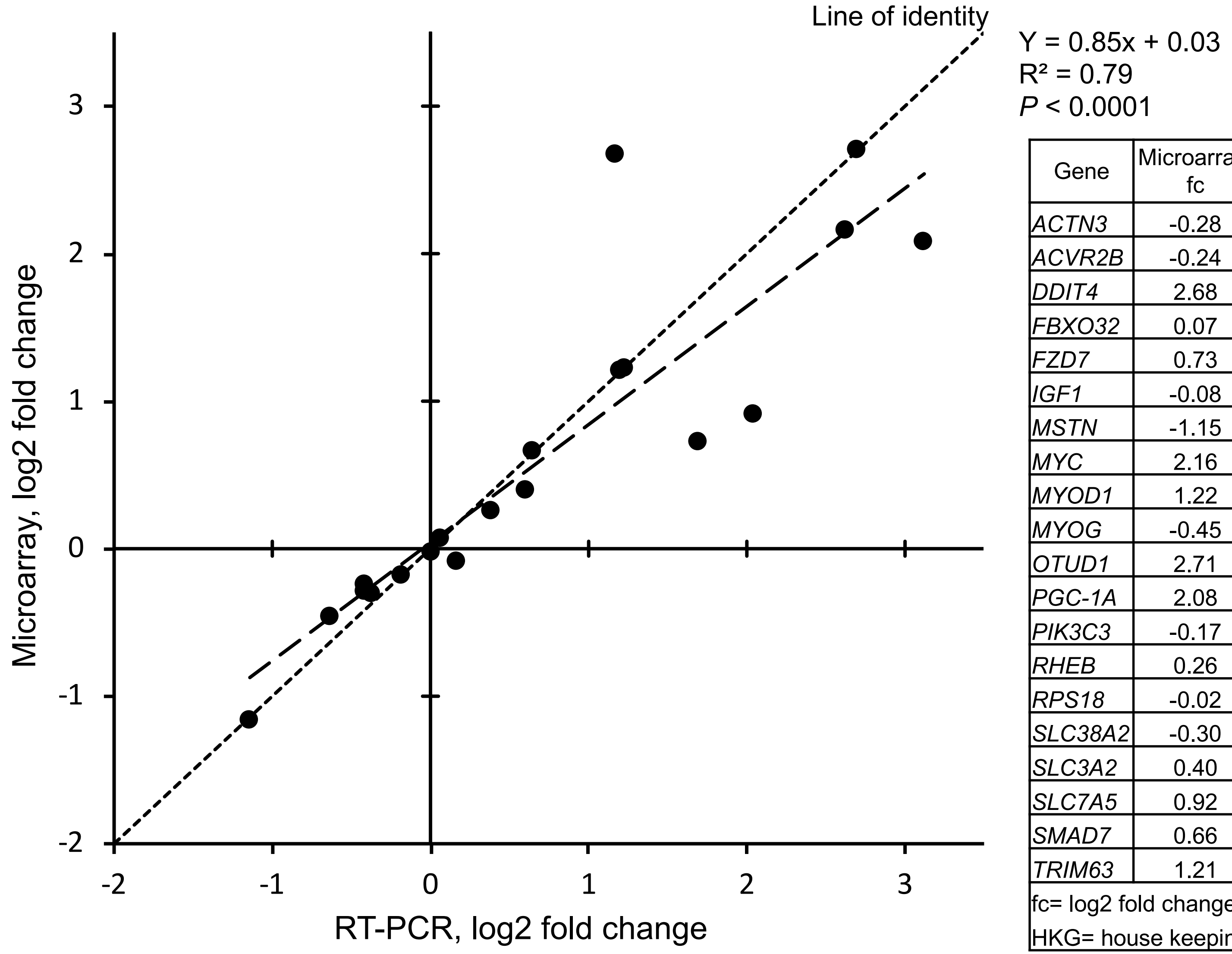


| Gene | Microarray, fc | RT-PCR, fc | RT-PCR, P-value |
|---|---|---|---|
| *ACTN3* | -0.28 | -0.42 | ns |
| *ACVR2B* | -0.24 | -0.41 | < 0.01 |
| *DDIT4* | 2.68 | 1.16 | < 0.05 |
| *FBXO32* | 0.07 | 0.06 | ns |
| *FZD7* | 0.73 | 1.70 | < 0.001 |
| *IGF1* | -0.08 | 0.17 | ns |
| *MSTN* | -1.15 | -1.14 | < 0.001 |
| *MYC* | 2.16 | 2.63 | < 0.001 |
| *MYOD1* | 1.22 | 1.23 | < 0.001 |
| *MYOG* | -0.45 | -0.63 | < 0.01 |
| *OTUD1* | 2.71 | 2.69 | < 0.05 |
| *PGC-1A* | 2.08 | 3.12 | < 0.001 |
| *PIK3C3* | -0.17 | -0.18 | < 0.1 |
| *RHEB* | 0.26 | 0.39 | < 0.01 |
| *RPS18* | -0.02 | 0.00 | HKG |
| *SLC38A2* | -0.30 | -0.37 | ns |
| *SLC3A2* | 0.40 | 0.60 | < 0.01 |
| *SLC7A5* | 0.92 | 2.05 | < 0.001 |
| *SMAD7* | 0.66 | 0.64 | < 0.001 |
| *TRIM63* | 1.21 | 1.19 | < 0.001 |

fc= log2 fold change
HKG= house keeping gene

**S3 Figure. Validation of the microarray by quantitative RT-PCR.** Correlation between sprint exercise-induced fold changes in gene expression (2 h and 20 min post-exercise compared with rest) analysed by microarray and RT-PCR. In the table, the coordinates for each dot (each gene) are given. *P*-values indicate the level of significance for the change in the gene expression as analysed by RT-PCR.
